# Left Thoracotomy as an Alternative to Redo Sternotomy in a Case of Left Ventricle Rupture

**DOI:** 10.7759/cureus.71833

**Published:** 2024-10-19

**Authors:** Salman Butt, Gaurav Pandey, Arun Kumar, Mitesh Badiwala, Umer Darr

**Affiliations:** 1 Cardiac Surgery, Cleveland Clinic Abu Dhabi, Abu Dhabi, ARE; 2 Cardiac Anesthesia, Cleveland Clinic Abu Dhabi, Abu Dhabi, ARE

**Keywords:** cardiopulmonary bypass, g6pd: glucose-6-phosphate dehydrogenase, left ventricular free wall rupture (lvfwr), st-elevation myocardial infarction (stemi), tee :transoesophageal echo

## Abstract

Left ventricular free wall rupture (LVFWR) is a rare but serious complication following ST-elevation myocardial infarction (MI), occurring in a small fraction of patients. Left Ventricular Free Wall Rupture presents as three types: There are three types of Left Ventricular Free Wall Rupture: Type 1 with an abrupt tear and high mortality, Type 2 with a slower tear, and Type 3 with aneurysm perforation. Despite reperfusion therapies, LVFWR remains concerning due to increased mortality described from 75% to 90%. We present a case of LVFWR in a 64-year-old with a history of previous aortic valve surgery and heart failure, managed through left thoracotomy surgical repair. Our approach led to successful repair, emphasizing collaborative intraoperative strategies for improved outcomes in LVFWR cases.

## Introduction

LVFWR is a rare but potentially life-threatening mechanical complication that can occur following ST-elevation myocardial infarction (MI). Recent studies have estimated that ventricular LVFWR may occur in a small fraction of 0.01% to 0.52% of patients who have experienced an MI. Morphologically, LVFWR was originally described in three forms: Type 1 rupture is an abrupt tear usually within the first 24 hours of MI and carries very high mortality and sudden cardiac death. Type 2 rupture is a slower tear with localized myocardial erosion, and Type 3 rupture is a thin-walled aneurysm perforation, which usually occurs more than 7 days after MI. A gradual and slow tear can be contained by thrombus formation or pericardial adhesions, with patients identified by symptoms and/or compromised hemodynamics. While reperfusion therapies have led to a decrease in the overall frequency of mechanical complications, LVFWR remains a significant concern due to its association with increased in-hospital mortality rates. Despite advances in medical care, there has been no substantial reduction in associated mortality rates over the past two decades for patients with LVFWR [1-5].

## Case presentation

**Figure 1:**
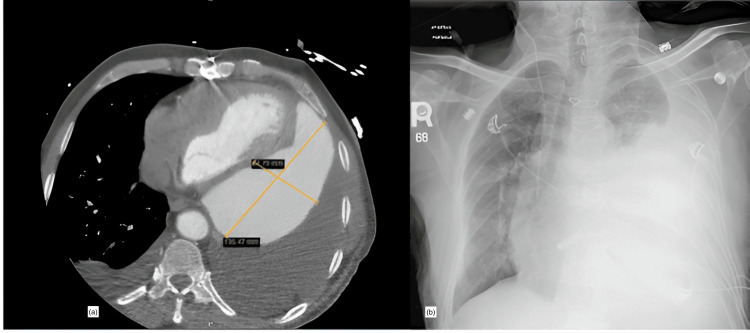
Axial views of the chest computed tomography scan reveal a contained left ventricle rupture and hemopericardium (a), and the PA view of the chest X-ray (b) reveal near total left lung compression secondary to a large serosanguineous left pleural effusion

A 64-year-old gentleman with a history of aortic valve replacement with a mechanical valve prosthesis and ascending aneurysm repair. The patient had a mechanical (St. Jude) Bentall procedure. He had a recent history of delayed presentation lateral wall ST-elevation myocardial infarction (STEMI), where the culprit coronary artery lesion in an obtuse marginal vessel was treated by primary coronary intervention (PCI), and he was discharged from the hospital. The patient presented to our facility with a one-month history of progressive dyspnea, orthopnea, and chest discomfort. He was found to have New York Heart Association (NYHA) class IV heart failure. His past medical history was also significant for hypertension, epilepsy, and glucose-6-phosphate dehydrogenase (G6PD) deficiency. He was also on warfarin and dual antiplatelet therapy for mechanical aortic valve and coronary artery disease, respectively. Due to the recent STEMI, the recommended is aspirin and clopidogrel. However, since he was on long-term warfarin (INR range 2.0 to 3.0), we routinely use clopidogrel and drop the aspirin to avoid the excessive bleeding risk. Further diagnostic investigations revealed an LVFWR with a 1.2 cm defect communicating freely with a large pseudoaneurysm of the left ventricle (Figure 1).

Urgent cardiac surgery was planned to repair the left ventricle (LV) via a left 4th intercostal space anterolateral thoracotomy approach with cardiopulmonary bypass support. The surgery was successfully performed, and the patient remained stable postoperatively, allowing for extubation within four hours after the procedure. The patient was discharged to the post-cardiac surgical ward on postoperative day 4 with stable hemodynamics and resolution of heart failure (NYHA class II).

## Discussion

In a manuscript by Gong and Nie, the authors present a novel clinical classification for ventricular free wall rupture (FWR) following acute myocardial infarction (AMI), delineating it into three distinct types: cardiac arrest, unstable, and stable. This classification is pivotal as it aids in the rapid identification and stratification of patients based on clinical severity, which can profoundly impact immediate management strategies and potentially enhance outcomes. The study underscores the necessity for swift and precise diagnostic and management approaches to address this life-threatening complication effectively. This classification could serve as a critical tool for clinicians, guiding the urgent surgical interventions that are often necessary to save lives, emphasizing the short window of opportunity for intervention, especially in the unstable type of FWR where timely surgical response could be life-saving [6].

A review by Sai Harika Pujari et al. discusses the role of extracorporeal membrane oxygenation (ECMO) in managing such critical cases, illustrating its value not only in treatment facilitation but also in extending the therapeutic window during the perioperative phase [7].

Matteo Matteucci et al. also highlighted the importance of prompt surgical intervention as a key to improving patient outcomes. Study analysis of several surgical techniques, including the use of prosthetic patches and sutureless methods, provides a comprehensive understanding that can guide clinical decisions in emergency cardiac care settings [8].

A case report by George H. Nasr demonstrates how delays in medical intervention for myocardial infarction during the COVID-19 pandemic led to severe complications such as left ventricular free wall rupture (LVFWR). Additionally, the study supports the use of point-of-care ultrasound in emergency settings to quickly identify conditions like LVFWR, enabling urgent surgical interventions critical for survival [9].

Intraoperative management strategy

Performing left ventricular free wall rupture (LVFWR) repair is a procedure associated with elevated risk, demanding close collaboration amongst the intraoperative team and a well-thought-out strategy. The integration of advanced diagnostic techniques also enhances clinical outcomes, emphasizing the importance of a cohesive approach in managing patients with this life-threatening condition. In this context, we outline the intraoperative management for three pivotal roles that synergistically led to a successful outcome for our patient [6].

Anesthesia management

**Figure 2:**
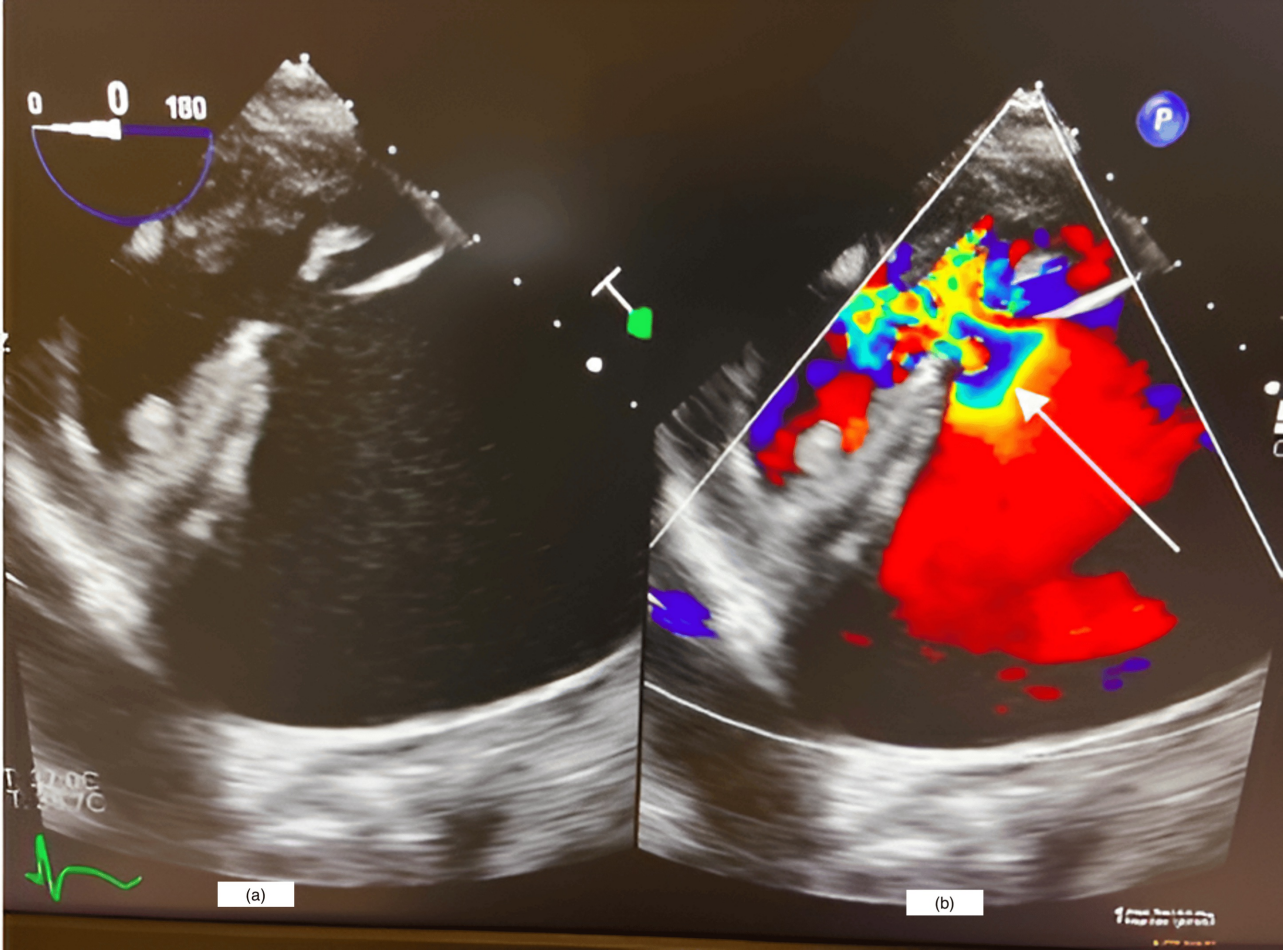
Intraoperative transesophageal echocardiography image showing left ventricular pseudoaneurysm

General anesthesia was planned to accommodate the left thoracotomy approach and peripheral cannulation for cardiopulmonary bypass (CPB). Anesthesia induction was uneventful, and the airway was secured with an 8.5-mm oral endotracheal tube. Single lung ventilation was achieved with a 9Fr Cohen bronchial blocker, and the position was confirmed by fiberoptic bronchoscopy. LV lateral free rupture was re-confirmed with a large pericardial and left pleural effusion on transoesophageal echocardiography (TEE). LV thrombus was ruled out as well. TEE-guided peripheral cannulation was done after full heparinization, and CPB was established. At the end of the surgery, the patient was successfully weaned from CPB, and he was transferred to the cardiac ICU in stable condition with minimal inotropic support (Figure 2, Video 1).

**Video 1:** Illustration of pre-repair-ECHO

Surgical management

Pre-operative analysis by echocardiogram and CT chest revealed that this was a contained free wall rupture. This occurred due to the adhesions from the prior aortic valve and ascending aortic aneurysm repair. This indeed represented an advantage for surgical planning. In our estimation, median sternotomy would have been challenging due to the posterior nature (circumflex artery STEMI) of the rupture. This would have necessitated substantial dissection and lifting/rotating of the heart to repair this injury. A thoracotomy obviated all these issues.

An 8 cm left thoracotomy was performed via the 4th intercostal space. Upon opening the left pleural space, 3 liters of dark serosanguinous fluid were drained and discarded. For CPB cannulation, 45,000 IU of unfractionated heparin was administered to achieve an activated clotting time (ACT) of >480 seconds. Arterial cannulation was performed using a 17-F cannula in the left common femoral artery, and a 25-F long venous drainage cannula was inserted through the common femoral vein. CPB was initiated, the heart was decompressed, and normothermia was maintained throughout.

**Figure 3:**
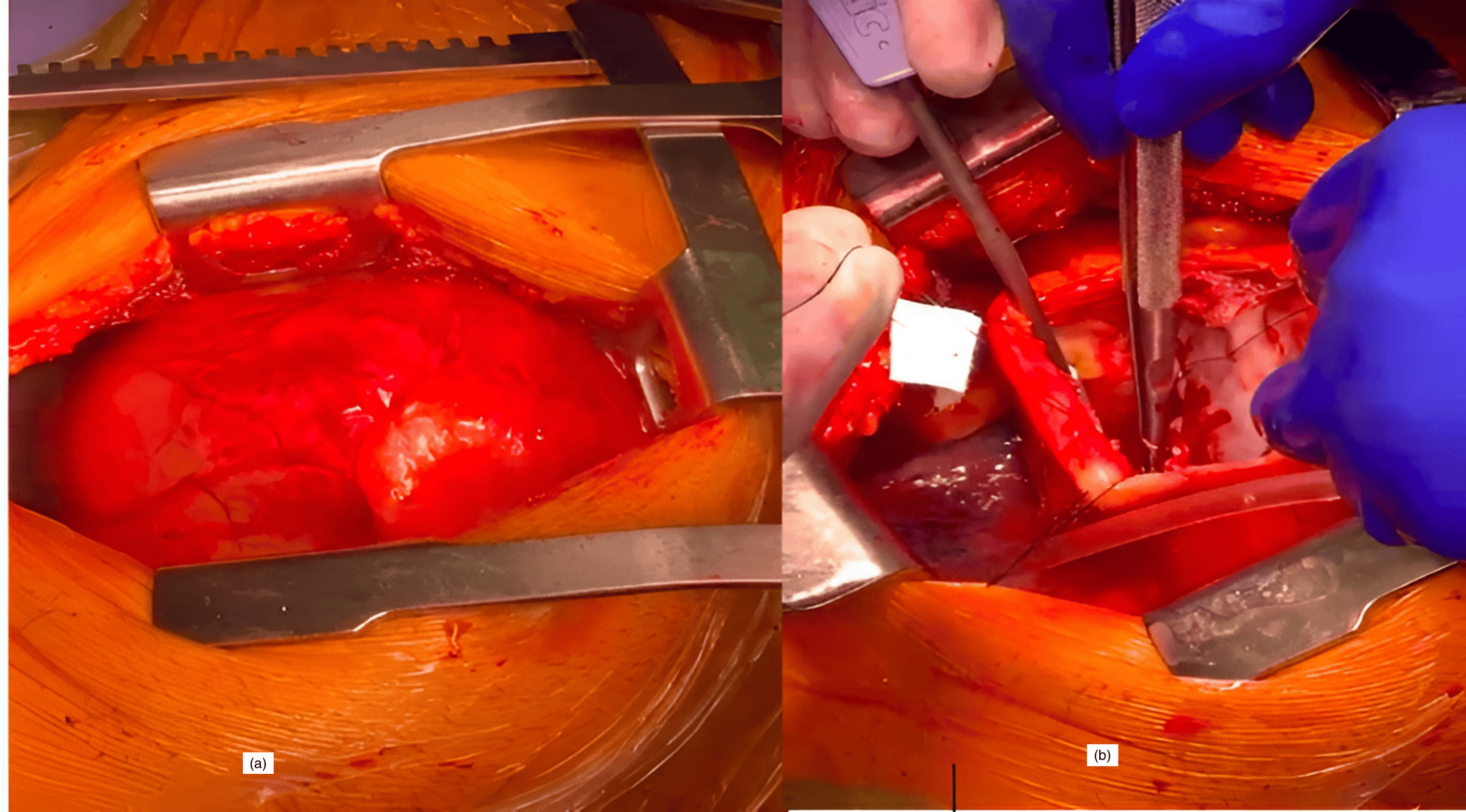
Illustration of the heart via left thoracotomy (a) and LV repair (b)

A tense pulsatile pericardium was encountered. Safe pericardial entry was limited by the proximity of the phrenic nerve. Pericardial stay sutures were placed, with special attention given to the identification and preservation of the phrenic nerve. Incising the pericardium further revealed fresh arterial bleeding, consistent with free-wall LV rupture (Figure 3). Repair of LV rupture was performed on the beating heart with pump assist. The heart was kept ejecting, preventing any risk of air embolization (albeit low risk). The additional advantage of a beating heart was the precise localization of the rupture zone. Post-STEMI rupture is rarely discretely punched out in the heart; especially in the acute phase, where there is a peri-infarct zone with several serpiginous bleeding points, in addition to muscle necrosis in the middle of the transmural infarction. Primary repair of the left ventricular rupture was performed using 2/0 prolene (MH needle) with felt strips for reinforcement.

After successful surgical repair, intra-op TEE confirmed an intact LV-free wall. CPB was weaned off with minimal inotropic support, and hemostasis was achieved after protamine reversal.

We believe that the left thoracotomy approach afforded several advantages. The exposure was superior to a midline redo-sternotomy. Bleeding from bone and mediastinal soft tissues was avoided. This approach was expeditious, avoided a potentially difficult mediastinal dissection, and permitted comprehensive assessment and drainage of the left hemithorax for additional sources of bleeding or pathology.

Perfusion management

At the outset of the procedure, the patient presented with anemia, characterized by a hemoglobin level of 89 g/l. To prevent severe hemodilution during CPB, one unit of red blood cells (RBCs) was employed in conjunction with retrograde and antegrade autologous prime techniques. During CPB initiation, utmost care was taken to ensure adequate flow while diligently maintaining the patient's normothermic state to optimize surgical conditions. Throughout the procedure, thorough attention was paid to maintaining adequate perfusion, leading to excellent gas exchange, cerebral saturations, and lactate levels. In this procedure, two cell savers were required due to the evacuation of 3 liters of dark serosanguinous fluid from the left pleural space upon its opening. The initial cell saver collected this fluid, but it was deemed prudent to discard it due to stagnation concerns and the intention of preventing any potential inflammatory reaction (Video 2).

**Video 2:** Illustration of post-repair-ECHO

## Conclusions

In summary, LVFWR is a rare but significant post-ST elevation myocardial infarction complication with persistent in-hospital mortality concerns. LVFWR can occur a few hours to days after MI but can also be the presenting complaint of a patient admitted to the emergency room. Keeping a high level of suspicion in mind, bedside echocardiography for MI patients presenting with cardiogenic shock can help diagnose the condition early. Successful management requires collaborative intraoperative strategies, including meticulous surgical repair, vigilant perfusion, and comprehensive anesthesia. The left thoracotomy approach offers advantages for redo sternotomy cases, and optimizing surgical conditions through adequate perfusion, anemia management, and normothermia is crucial. This case emphasizes the importance of interdisciplinary teamwork and strategic planning to improve LVFWR patient outcomes.
